# Preliminary Genetic Analysis Supports Cave Populations as Targets for Conservation in the Endemic Endangered Puerto Rican Boa (Boidae: *Epicrates inornatus*)

**DOI:** 10.1371/journal.pone.0063899

**Published:** 2013-05-15

**Authors:** Alberto R. Puente-Rolón, R. Graham Reynolds, Liam J. Revell

**Affiliations:** 1 Departamento de Ciencias y Tecnología, Universidad Interamericana de Puerto Rico, Recinto de Arecibo, Arecibo, Puerto Rico; 2 Department of Biology, University of Massachusetts Boston, Boston, Massachusetts, United States of America; Smithsonian’s National Zoological Park, United States of America

## Abstract

The endemic Puerto Rican boa (*Epicrates inornatus*) has spent 42 years on the Endangered Species List with little evidence for recovery. One significant impediment to effective conservation planning has been a lack of knowledge of the distribution of genetic variability in the species. It has previously been suggested that boas might best be protected around caves that harbor large populations of bats. Prior study has found Puerto Rican boas at relatively high densities in and around bat caves, which they use both to feed and seek shelter. However, it is unknown whether these behaviorally distinctive populations represent a distinct evolutionary lineage, or (conversely) whether caves harbor representative genetic diversity for the species across the island. We provide the first genetic study of the Puerto Rican boa, and we examine and compare genetic diversity and divergence among two cave populations and two surface populations of boas. We find three haplogroups and an apparent lack of phylogeographic structure across the island. In addition, we find that the two cave populations appear no less diverse than the two surface populations, and harbor multiple mtDNA lineages. We discuss the conservation implications of these findings, including a call for the immediate protection of the remaining cave-associated populations of boas.

## Introduction

**Figure 1:**
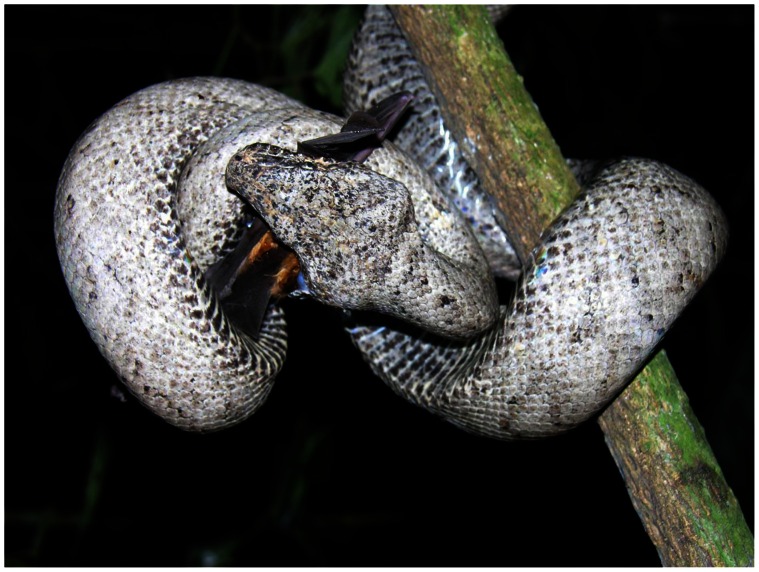
A hypomelanistic endemic endangered Puerto Rican Boa (*Epicrates inornatus*) consuming a bat (*Monophyllus redmani*), Mata de Plátano Cave, Arecibo municipality. Photo by ARPR.

In the Caribbean, the boid genus *Epicrates* represents a diverse and ecologically important group of snakes comprising ten or more endemic species– at least three in the Bahama Archipelago and at least seven in the Greater Antilles [1]. The Puerto Rican boa (*E. inornatus*) [Fig. 1], endemic to the main island of Puerto Rico, was declared endangered in 1970 by the United States Fish and Wildlife Service (Endangered Species Act 1973) and a recovery plan was implemented in 1986 [2]. In 2004, the Puerto Rico Department of Natural and Environmental Resources classified this species as vulnerable, though it is still considered an endangered species by the U. S. Fish and Wildlife Service. A subsequent 5-year evaluation was completed in 2011. This evaluation determined that the species is both sufficiently in danger of extinction, and missing data relevant to recovery criteria, to recommend that it not be down-listed [3].

More than four decades after the designation of *Epicrates inornatus* as endangered, and in spite of some meaningful initial studies (e.g., [4], [5], [6], [7]), many important aspects of the ecology, natural history, and, especially, the genetics of this species are not well characterized. The species still faces a range of threats, including habitat loss, road construction and road mortality, invasive species, malicious killing, and illegal trade; while a lack of information regarding the extent and distribution of genetic variation within the species has hampered conservation strategies [3]. The use of population genetics in initial conservation assessment and planning can be a powerful tool for devising conservation strategies that minimize the disturbance to the evolutionary trajectories for island populations [8]. When defining conservation strategies for endangered species, it is also important to recognize that separate evolutionary-significant units (ESUs; [9], [10]) might exist, particularly on islands with high habitat heterogeneity or potential barriers to gene flow, such that genetically differentiated populations should be managed separately while genetically similar populations might be managed jointly (e.g., [11]). Though West Indian boids are an imperiled group [12], conservation genetic studies have been conducted for only two other species of West Indian boas: the Turks Island boa (*E. chrysogaster*; [11]) and the Jamaican boa (*E. subflavus*; [13]). In both these cases, genetic study led to an improved understanding of the phylogeographic patterns and units of conservation for these species.

**Figure 2:**
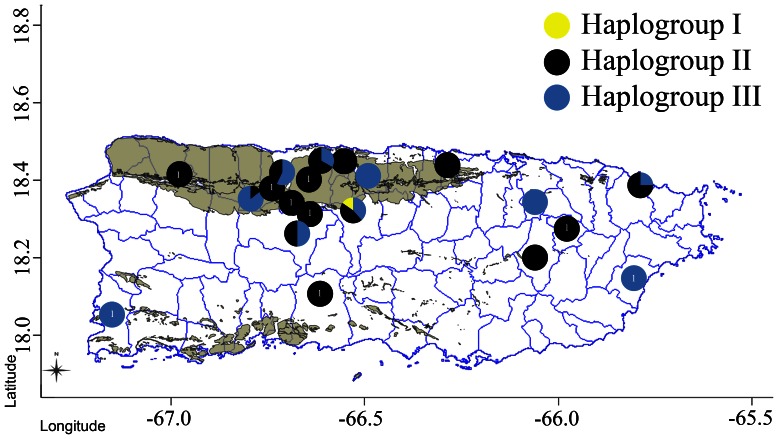
Map of Puerto Rico overlaid with the karst region (brown) and mtDNA haplogroups found in the Puerto Rican Boa, mapped by sampling locality on the island. Sampling locations with a single individual are labeled with a “1.” Note that no distinct phylogeographic signal is found, and that Haplogroup I appears restricted to a single population.

Puerto Rico is the smallest of the four main islands of the Greater Antilles, with a total land area of 8900 km^2^, but is characterized by high habitat heterogeneity over relatively short distances. Much of northwestern Puerto Rico is geologically distinguished by a region of limestone known as the karst belt, covering nearly 1426 km^2^ (Fig. 2). This region encompasses many of Puerto Rico’s most important natural resources, including the most extensive freshwater aquifer and the largest tract of mature forest on the island; as well as unique geological and ecological features such as cave systems and isolated towers of karst known as “haystack hills” or “mogotes” [14]. The region is also characterized by high ecological diversity, including the highest diversity of tree species in Puerto Rico, and is a vital habitat for many species of conservation concern [14]. Importantly, the forests of the karst region are considered crucial to Puerto Rican boas, as the densest populations of boas are reported from this region [15], [16] and many populations exhibit unique ecological adaptations to cave use [6]. Recently it has become apparent that local populations of boas routinely exploit bat caves, which represent a unique centralized food resource [6]. Though usually solitary, dozens of boas are known to congregate around the mouths or inside of caves where they use tactile feeding to capture bats from the air [17], [18]. Furthermore, boas, especially large females, are also known to use caves as refugia, to which they are presumably attracted by the relative safety from predators and constant ambient heat and humidity. Because cave-associated populations of boas tend to have smaller home ranges than “surface” populations [6], [7], caves potentially represent tractable units of conservation, whereby the protection of caves and surrounding habitat protects a dense population of boas and their food resource. To examine genetic diversity within and between behaviorally differentiated populations of boas, we genetically sampled boas from two cave and two surface populations in the karst region of Puerto Rico, as well as boas across the island of Puerto Rico. Using both mitochondrial and microsatellite data we examine phylogeographic relationships across the island, as well as intra- and inter- population genetic diversity and divergence among our cave and surface populations of boas. We also discuss our findings in the context of conservation for this species.

## Materials and Methods

### Ethics Statement

All samples were collected under DRNA permits 2012-EPE-001 (to RGR) and 00-EPE-16 (to ARPR), U.S. Fish and Wildlife Native Endangered Species Recovery Permit # TE63270A-0 (to RGR), and U.S. Department of Agriculture Special Use Permit # CNF-2118 (to RGR). This work was approved by the University of Massachusetts Boston Institutional Animal Care and Use Committee (IACUC) Protocol no. 2011006.

### Sample Collection and DNA Extraction

We collected samples of *E. inornatus* from two cave populations: Agrodel Cave (Hatillo) and Mata de Plátano Cave (Arecibo), and two surface populations: Dorado Beach (Dorado) and Río Encantado (Ciales). We chose these populations as they are some of the few in the karst region where animals can be regularly found. We sampled populations through both focused sampling (cave and surface) and opportunistic sampling (surface only). Focused sampling in the cave populations consisted of one or more of the authors searching the cave mouth during the day or at night using flashlights. Boas around the cave mouths were often found coiled in trees during the day and hanging from vines or the cave wall at night while foraging for bats exiting the cave. Nocturnal searches were usually concluded before midnight, after which time boa activity drops off [6]. Cave samples were also obtained by entering the caves diurnally to search for boas using the cave as a refugium or feeding on bats in the cave interior. The authors and other individuals from speleological societies trained in cave exploration conducted these searches, as entering these caves requires advanced caving skills. Boas within the caves were found coiled on the ground, on cave walls and ceilings, or actively foraging near bat colonies. All cave boas were individually marked with a passive integrated transponder (PIT) tag for subsequent identification as part of another ongoing study. We sampled surface populations using focused nocturnal sampling of appropriate habitat on foot. We also obtained samples opportunistically from live and dead boas while driving along roads within the two surface populations. Finally, we used both focused and opportunistic sampling to obtain samples from boas across the island of Puerto Rico between April 2001 and March 2012.

Samples from boas consisted of 3–10 mm tail clips (live specimens) or dissected muscle tissue (road killed specimens) preserved in 95% ethanol. We sanitized tails before and after clipping and applied antiseptic dermal adhesive to prevent infection. Any boa found with a clipped or damaged tail tip was not sampled to prevent repeated sampling. We extracted whole genomic DNA using the Promega Wizard SV DNA purification system according to the manufacturer’s protocol and stored the extracts at −20°C.

### Mitochondrial DNA Sequencing

We used the polymerase chain reaction (PCR) to amplify two fragments of the mitochondrial genome: cytochrome B (*CYTB*; primers and conditions in [19] and NADH dehydrogenase subunit 4 (*ND4*; primers and conditions in [20] in reactions conducted in an Eppendorf Mastercycler. We visualized PCR products by gel electrophoresis and purified and sequenced products on an automated sequencer (ABI 3730XL) at Massachusetts General Hospital DNA Core Facility, Cambridge, MA. We assembled contigs and manually verified ambiguous base calls using Sequencher 5.0 (Gene Code). We then aligned sequences using the ClustalW 2.1 algorithm [21] implemented in Mesquite 2.75 [22] using reference sequences. We deposited all sequences in GenBank (Accessions KC819418-KC819589).

### Mitochondrial DNA Analyses

We concatenated the two mitochondrial gene fragments and created a statistical parsimony network using default parameters (95% probability criterion) in the program TCS 1.21 [23]. We estimated genetic variation within and across populations as nucleotide (π) and haplotype (*h*) diversity using Arlequin 3.5 [24], and we used Tajima’s D and Fu’s Fs as tests for mutation-drift equilibrium in Arlequin. To investigate partitioning of genetic variation across Puerto Rico we calculated *Φ*-statistics in an analysis of molecular variance (AMOVA) framework [25] for various groupings of populations, including within and between karst and non-karst ecoregions, as well as within and between cave and surface populations of *E. inornatus*. Generally, use of AMOVA is not recommended for fewer than seven groups due to the inability to achieve strict statistical significance for the parameter *Φ_CT_* [26]. Consequently, we consider our use of the AMOVA on fewer than seven distinct groups to be a heuristic approach to assess the partitioning of genetic variability in our dataset. Finally, we estimated population pairwise *Φ_ST_* in Arlequin. Significance of *Φ_ST_* values was determined via the maximum number of permutations in Arlequin 3.5.

### Microsatellite Genotyping

As no species-specific markers exist for *E. inornatus*, we initially screened a subset of five individuals at 20 microsatellite loci developed for other boid snakes, specifically *E. subflavus* (*usat*-1, *usat*-3, *usat*-10, *usat*-11, *usat*-13, *usat*-16, *usat*-20, *usat*-24, *usat*-30, and *usat*-36) [27], *usat*-2, *usat*-4, *usat*-6, *usat*-14, and *usat*-32 [28], and *Boa constrictor imperator* (*Bci*-14, *Bci*-15, *Bci*-18, *Bci*-21, *Bci*-23) [29]. We labeled primer pairs amplifying products with one of four dyes (6-FAM, PET, VIC, or NED) on the 5′ end of the reverse primer, and then genotyped all five test samples at each locus. We resolved genotypes on the above sequencing equipment using GeneScan™ 500 LIZ size standard and Peak Scanner 1.0 software (ABI) with manual verification of peak calling. We used all polymorphic loci with consistent peak calling within the expected size range to genotype individuals from two cave populations (Agrodel Cave and Mata de Plátano Cave) and two surface populations (Dorado Beach and Río Encantado). We tested for genotyping errors by randomly selecting 50% of the samples for repeated genotyping from the PCR stage. In addition, we used Micro-Checker 2.2.3 [30] to investigate whether our genotype profiles showed evidence of allele-dropout or null alleles.

### Microsatellite Analysis

We calculated the number of alleles (N_A_), effective number of alleles (N_E_), observed heterozygosity (H_O_), and expected heterozygosity (H_E_) using GenAlEx 6.4 [31]. We also visualized genotypic divergence in multivariate space using principal components analysis (PCA) implemented in GenAlEx. We estimated the inbreeding coefficient within populations (*F_IS_*) in GenePop 4.0 [32], and we tested for departures from Hardy–Weinberg equilibrium (HWE) and genotypic differentiation between populations using exact tests with 10,000 dememorizations, 2,500 batches, and 20,000 iterations per batch implemented. We conducted an AMOVA for cave and surface populations, and estimated population pairwise *Φ_ST_* in Arlequin. We computed estimates of the p-values of each *Φ_ST_* value via the maximum number of permutations in Arlequin 3.5.

## Results

We obtained a total of 86 samples from 15 municipalities across Puerto Rico (Fig. 2), a comparable sample size to other studies of West Indian Boas (n = 87 [13]; n = 53 [11]), and an unprecedented genetic sample for Puerto Rican boas. Twenty one and seven samples were obtained from each of the two cave populations (Agrodel and Mata de Plátano, respectively), and nine and 15 samples were obtained from the two surface populations (Dorado Beach and Río Encantado, respectively). Over 11 years, we obtained two or more samples (range 2–6; avg. 3.1) from eight additional populations in seven municipalities, and a single sample from nine populations in a further six municipalities (Table 1).

**Table 1:** Locality information, number of samples, and sampling type(s) for cave, surface, and all other populations of *Epicrates inornatus* sampled from Puerto Rico.

Population	Municipality	# Samples	Samplingtype(s)
*Cave*			
Mata de Plátano Cave	Arecibo	7	Focused
Agrodel Cave	Hatillo	21	Focused
*Surface*			
Río Encantado	Ciales	15	Focused/Opportunistic
Dorado Beach	Dorado	9	Focused
*Others*			
Carretera 123	Arecibo	1	Opportunistic
Bosque Bello Station	Arecibo	1	Opportunistic
Río Abajo	Arecibo	1	Focused
Cambalache State Forest	Arecibo	6	Opportunistic
Miraflores	Arecibo	1	Opportunistic
Arecibo	Arecibo	4	Opportunistic
Barceloneta	Barceloneta	2	Focused
Cabo Rojo	Cabo Rojo	1	Opportunistic
Caguas	Caguas	2	Opportunistic
Cupey	San Juan	2	Opportunistic
Gurabo	Gurabo	1	Opportunistic
Humacao	Humacao	1	Opportunistic
Guajataca	Isabela	1	Focused
Sabana Seca	Manatí	3	Focused
Ponce	Ponce	1	Opportunistic
Cerro El Faro	Río Grande	4	Focused
Utuado	Utuado	2	Opportunistic

See methods for details on sampling.

### Mitochondrial DNA Analyses

**Figure 3:**
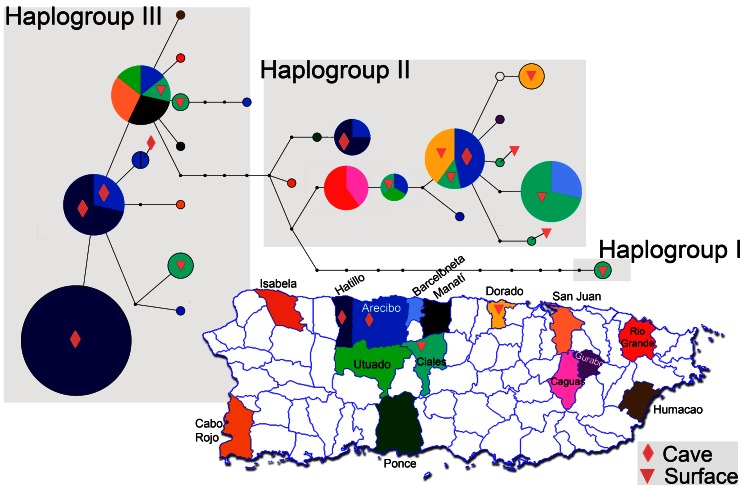
Map of Puerto Rico, where municipalities with samples of *Epicrates inornatus* are individually colored. A haplotype network is shown above the map, with each haplotype color-coded by the proportion of individuals from each municipality represented in the haplotype. Haplotype circles are sized proportionally to the number of individuals represented in each haplotype, where the smallest circles represent one individual and the largest represents 13 individuals. Line segment lengths are arbitrary, and the smallest black circles represent un-sampled mutational steps. Three haplogroups are recovered and separated into gray boxes. Two cave (Agrodel [Hatillo], and Mata de Plátano [Arecibo]) and two surface (Río Encantado [Ciales], and Dorado Beach [Dorado]) populations are labeled both on the map and on the haplotype network.

We obtained a total of 1754 base pairs (bp) (1110 bp CYTB [complete cds]; 644 bp ND4 [partial cds]) of mtDNA sequence from each of 86 individual *E. inornatus* from 15 municipalities across Puerto Rico. A haplotype network constructed from the concatenated dataset identified three clear haplogroups. Haplogroup I consisted of a single haplotype (two individuals) separated by a minimum of 12 mutational steps from Haplogroup II (13 haplotypes, 44 individuals), which is in turn separated by a minimum of five mutational steps from Haplogroup III (12 haplotypes, 42 individuals) (Fig. 3). No distinct phylogeographic pattern emerges when these haplogroups are mapped onto their sampling localities (Fig. 2). Haplogroups II and III were both found in each cave and surface focal population except for Dorado Beach, which contained only two haplotypes of Haplogroup II (Fig. 3). Haplogroup I was found in only two individuals from a single population (Río Encantado).

**Figure 4:**
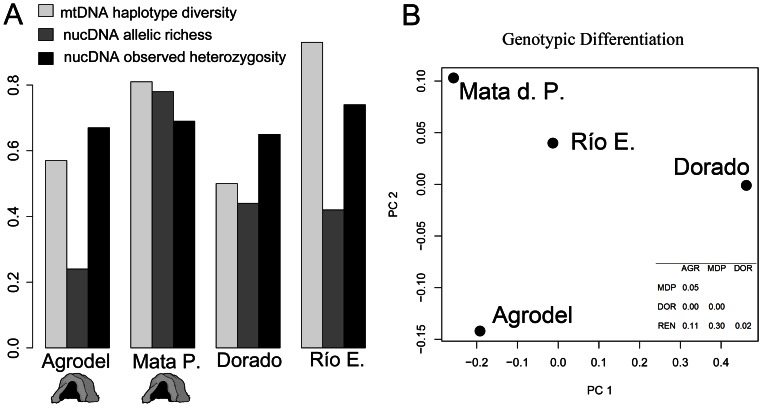
Plots of genetic diversity for cave and surface populations of Puerto Rican boas. A) mtDNA and nucDNA estimates for each cave and surface population. The parameters are haplotype diversity (*h*), observed heterozygosity (H_O_), and allelic richness (A). B) Genotypic differentiation among populations visualized in multivariate space using principal components rotations. Inset: *P*-values for Fisher’s tests for genetic differentiation between populations.

In cave populations, we found three haplotypes (2 private) in Agrodel and 2 haplotypes (1 private) in Mata de Plátano. Similarly, the Dorado Beach surface population had two haplotypes (1 private), while Río Encantado had nine haplotypes (5 private). Pairwise genetic (haplotype) diversity was more similar between any cave and surface population than between both cave populations or both surface populations, with Agrodel cave (*h* = 0.57) and Dorado Beach (*h* = 0.50) having relatively lower amounts of haplotypic diversity than Mata de Plátano (*h* = 0.81) and Río Encantado (*h* = 0.93) [Table 2, Fig. 4A]. Overall haplotypic diversity in cave and surface populations was *h* = 0.92, and we found no evidence for mutation-drift non-equilibrium in any population (Table 2). AMOVA analyses revealed that in comparisons of karst and non-karst populations and cave and surface populations, the majority of the genetic variance is observed within populations (79.8%, *Φ_ST_* = 0.20; and 51.8%, *Φ_ST_* = 0.48 respectively), rather than between them (Table 3). Nonetheless, pairwise comparisons (*F_ST_*) between populations revealed significantly non-zero divergence between all population pairs except for between Mata de Plátano (cave) and Río Encantado (surface; *F*_ST_ = 0.005; *P* = 0.35) [Table 4].

**Table 2:** Genetic diversity and test statistics of selective neutrality with populations of *Epicrates inornatus* for the concatenated mitochondrial dataset.

Population	S	*n*	K	*h*	Π	Tajima’s D	Fu’s FS
*Cave*							
Agrodel	10	3	2	0.57	0.00160	0.04	4.69
Mata de Plátano	13	2	1	0.81	0.00370	1.31	2.35
*Surface*							
Dorado Beach	2	2	1	0.50	0.00057	1.23	2.08
Río Encantado	27	9	5	0.93	0.00500	0.29	0.57
*All*	45	26	20	0.92	0.00399	−0.69	−3.66

S: number of segregating sites, *n*: number of haplotypes, K: number of private haplotypes, *h*: haplotype diversity, π: nucleotide diversity. Significance for neutrality tests were based on 10,000 permutations with α = 0.05.

**Table 3:** Results of analysis of molecular variance (AMOVA) for various groupings of *Epicrates inornatus* for both mtDNA and microsatellite data.

Marker	Source of Variation	d.f.	Variance component	% total variance	Φ statistics	*P*
mtDNA	Karst/Non-Karst	1	−0.4	−11.1	Φ_CT_ = −0.11	0.67
	Among populations	5	1.1	31.3	Φ_SC_ = 0.28	<0.001
	Within populations	86	2.7	79.8	Φ_ST_ = 0.20	<0.001
	Cave/Surface	1	1.4	31.3	Φ_CT_ = 0.31	0.33
	Among populations	2	0.8	16.9	Φ_SC_ = 0.25	<0.001
	Within populations	48	2.4	51.8	Φ_ST_ = 0.48	<0.001
µsats	Cave/Surface	1	0.01	1.86	Φ_CT_ = 0.02	0.33
	Among populations	2	0.02	3.23	Φ_SC_ = 0.03	0.06
	Within populations	98	2.4	94.9	Φ_ST_ = 0.05	0.002

**Table 4:** Pairwise genetic divergence between sampling locations (cave and surface populations only).

	AGR	MDP	DOR	REN
AGR		0.27*	0.79*	0.39*
MDP	0.038		0.48*	0.005
DOR	0.11*	0.10*		0.23*
REN	0.017	0.014	0.028	

Above diagonal: mtDNA distances (*Φ_ST_*); below diagonal, microsatellite distances (*F_ST_*). P-values (* significant at *P*<0.05) were obtained through 99,999 permutations.

### Microsatellite Analysis

We screened a total of 20 microsatellite loci. Of the 12 that we successfully amplified (*Bci*-15, *Bci*-23, *usat*-1, *usat*-2, *usat*-3, *usat*-4, *usat*-16, *usat*-20, *usat*-24, *usat*-30, *usat*-32, and *usat*-36) three showed repeatable polymorphism (*usat*-1, *usat*-3, and *usat*-32). We obtained genotypes at these three loci for a total of 51 individuals from two cave (Agrodel cave [n = 21] and Mata de Plátano cave [n = 5]) and two surface populations (Dorado Beach [n = 9] and Río Encantado [n = 13]). We found a total of 21 alleles across the three loci among 51 individuals from the cave and surface populations. We found no evidence of null alleles or allele dropout, and the scoring error rate was 0.013 errors/locus (2 errors across the entire dataset). Exact tests indicated that all loci were in HWE (Table 5). Allelic richness ranged between 0.24 (Agrodel cave) and 0.78 (Mata de Plátano cave) [Table 6, Fig. 4A]. Each cave population had a single private allele, while the surface population Dorado Beach had none and Río Encantado had two. Expected heterozygosity, H_E,_ ranged between 0.65 (Dorado Beach) and 0.74 (Río Encantado) [Fig. 4A]. One population (Mata de Plátano cave) showed an elevated inbreeding coefficient (F_IS_ = 0.2) indicating potential non-random mating or declining population size in this population. Estimates of genotypic differentiation between populations revealed significant divergence between Dorado Beach and the other three populations (Río Encantado, *P* = 0.02; Agrodel cave, *P*<0.0001; and Mata de Plátano cave *P*<0.0001), as well as between Agrodel cave Mata de Plátano cave (*P* = 0.05) [Fig. 4B]. Pairwise comparisons (*F_ST_*) between populations revealed low but significant divergence between Dorado Beach and Agrodel cave (*F_ST_* = 0.11; *P* = 0.004), as well as Dorado Beach and Mata de Plátano cave (*F_ST_ = *0.10; *P* = 0.008). Like the AMOVA analyses for mtDNA, in comparisons of cave and surface populations, the majority of the genetic variance is observed within populations, rather than among them (94.9%, *Φ_ST_* = 0.005).

**Table 5:** Microsatellite loci used to genotype samples in this study.

Locus	Repeat Motif	Size Range	N_A_	HWE
µsat-1	(AGAT)^n^	313–337	7	0.20
µsat-3	(TCCA)^n^	220–240	6	0.87
µsat-32	(ATC)^n^	358–379	8	0.48

N_A_, number of alleles per locus; HWE, *P*-value for test of Hardy-Weinberg equilibrium.

**Table 6:** Sample sizes and summary statistics of microsatellite gene diversity (SE) for the two cave and two surface populations.

Population	*n*	N_A_	N_E_	H_O_	H_E_	PA	A	F_IS_
*Cave*								
Agrodel	21	5.0	3.16	0.71 (0.09)	0.67 (0.04)	1	0.24	−0.02
Mata de Plátano	7	4.7	3.27	0.61 (0.06)	0.69 (0.02)	1	0.78	0.2
*Surface*								
Dorado Beach	9	4.0	3.02	0.63 (0.10)	0.65 (0.06)	0	0.44	0.09
Río Encantado	15	6.3	4.20	0.89 (0.06)	0.74 (0.04)	2	0.42	−0.16

N_A_, number of alleles; N_E_, number of effective alleles; H_O_, mean observed heterozygosity; H_E_, mean expected heterozygosity; PA, number of private alleles (frequency); A, mean allelic richness adjusted for sample size; F_IS_, mean level of inbreeding observed.

## Discussion

### Caves as Units of Conservation

It has been suggested that cave-associated populations of boas might represent tractable conservation priorities, as these populations are denser and individuals tend to have smaller home ranges around caves [6], [7], and habitats surrounding caves are frequently protected for recreational use and cultural value [33]. A single cave may harbor more than 50 boas at any given time, while animals in surface populations are likely more dispersed and would require a much greater area to encapsulate the ranges of the same number of snakes [34]. Our data show that genetic diversity in two cave populations is similar to surface populations (Table 2), and includes individuals of highly divergent mtDNA haplogroups which are also found across the island of Puerto Rico (Figs. 2, 3). Although most cave and surface populations showed significant divergence, the amount of divergence was slight and AMOVA indicated that the majority of genetic variance is found within, not between, populations. These data indicate that conserving a single cave population would preserve multiple genetic lineages, which represents a large proportion of the genetic diversity of Puerto Rican boas. In addition, conserving multiple cave populations would greatly increase the amount of genetic diversity being protected, as cave populations do not appear to be any more similar genetically than cave and surface populations in the same region.

For microsatellite data, we found that cave populations contained private alleles and allelic richness that encompassed both the low (A = 0.24, Agrodel) and high (A = 0.78, Mata de Plátano) estimates for the four populations. High allelic richness might be expected if there is a high level of gene flow, though in the Mata de Plátano cave population this is contradicted by estimates of an inbreeding coefficient (*F_IS_* = 0.2) that suggests non-random mating. Since our sample from Mata de Plátano was relatively small, more samples from this cave are necessary to reveal at what sampling level allelic richness asymptotes and yield better estimates of population genetic parameters, including better estimates of population structure. However, other work suggests that the population of boas at this cave has been declining in recent years [6] making sampling unique individuals increasingly difficult. Observed heterozygosity was similar for all populations. Like our results for mtDNA, AMOVA analyses showed that the vast majority of genetic variation in the microsatellite data (95%) is explained by within-population grouping, indicating that individual populations harbor a relatively high level of genetic diversity. The Dorado Beach population was significantly differentiated from other populations (Table 4, Fig. 4B), a situation which may be consistent with isolation-by-distance. An alternative explanation is that this population, while directly adjacent to the karst region of Puerto Rico, exists in a highly fragmented landscape of a largely developed municipality. Hence it is possible that this population is experiencing the effects of genetic drift due to the barriers to gene flow created by human encroachment. In multivariate space, cave populations are significantly differentiated from each other as revealed by exact tests (Fig. 4B), though not from the surface Río Encantado population.

Overall, we find no evidence for genetic differentiation between cave and non-cave populations, indicating that there is no evidence for a “cave exploiting lineage” of Puerto Rican boas. Indeed, it appears that the remarkable behavioral adaptation of bat predation may be a plastic trait; though future studies would be required to verify this assertion.

### Conservation Genetics of West Indian Boas

Conservation genetic and phylogeographic approaches have been used in only two other species of West Indian boas: the Turks Island boa (*Epicrates chrysogaster*; [11]) and the Jamaican boa (*E. subflavus*; [13]); each of which has yielded significant information regarding the distribution of genetic variation in these species. For instance, Reynolds *et al.* [11] found relatively low genetic divergence in Turks Island boas across the Turks and Caicos archipelago, a finding with significant conservation and management implications. These authors suggested that the Turks Island boa represents a single evolutionarily significant unit, and therefore recommended that translocation and reintroduction campaigns would not disturb any significant genetic structure, a strategy that may well be the most important component of long-term conservation management of this species [11], [35]. Tzika *et al.* [13] identified relevant geographical management units in the Jamaican boa, suggesting that populations in the Blue and John Crow Mountains be managed separately as a unique lineage and that the population in Cockpit country be given a much higher level of protection than is currently afforded in practice.

Combined with the present study, a clearer picture is emerging regarding the extent of genetic diversity and phylogeographic structure in West Indian boas. In the Turks and Caicos archipelago, rising sea level since the height of the Wisconsin glaciation has resulted in the separation of boas across a shallow bank (Caicos) as well as across a deep-water channel (Turks Island Passage) [36]. However, very little divergence was found among these populations, a finding which Reynolds *et al.* [11] suggest could be owing to generally low effective population sizes and a lack of historic population structure (high gene flow across the emergent banks). In Jamaican boas, two haplogroups were found, roughly corresponding to an Eastern montane lineage and a western+central lineage. However, very little divergence was observed in the mtDNA haplotypes, with most intragroup haplotypes being separated by a single polymorphism and the two haplogroups being separated by a minimum of only six mutational steps [13]. It is important to note that Jamaica has had a complex geologic history, including complete submergence until the Miocene and formation through the amalgamation of three proto-island blocks [37].

In addition, a study of the phylogenetics and biogeography of *Epicrates* suggests that the origins of diversity are from dispersal among West Indian islands and island banks, with little evidence for intra-island speciation (Reynolds *et al.* unpublished data). Although these authors did not include one species with potential bearing on this matter, *E. gracilis*, which might have evolved from peripatric speciation within the island or proto-islands of Hispaniola, it is notable that there is no evidence for speciation even in large and heterogeneous islands such as Cuba. These results, combined with intraspecific studies (including the present study) showing little phylogeographic structure, might suggest that boas are vagile and have elevated genetic connectivity (gene flow) over fairly broad geographic areas. This is in stark contrast to other West Indian squamates, such as *Anolis* lizards. For instance, in the widespread Puerto Rican mountain garden lizard (*A. krugi*), although fine and broad scale connectivity exists in this species, there is a high degree of genetic structure and isolation by distance across larger areas in the apparent absence of any obvious barriers to gene flow [38]. Extremely high population sizes and low vagility (owing to territoriality and philopatry) in anoles likely contribute to a much lower degree of gene flow across distances greater than a kilometer, a distance readily traveled by an individual Puerto Rican boa [6].

In Puerto Rico, boas appear to exhibit little to no phylogeographic structure, as the main haplogroups are distributed across the island with no measurable partitioning by ecoregion (e.g. karst, rainforest, etc.) or across the central mountain ranges (Sierra de Luquillo, Cordillera Central). Indeed, in the four focal populations, at least two haplogroups can be found in each population. Prior to recent anthropogenic deforestation and disturbance, it is likely that boas consistently occupied almost all habitats on the island below about 500 m elevation, though they are occasionally found at higher elevations as well [1], [3]. This suggests that the mountain ranges would not necessarily have represented barriers to gene flow. In addition, boas have been subjected to a systematic relocation effort over the last 40 years or so (ARPR pers. ob.), whereby “nuisance” boas are removed from homes and farms and relocated to forested areas. While the extent of this practice has not been quantified, it is extremely common, and may also account for some mixture of haplotypes across the island.

### Conclusions

Puerto Rican boas are currently known from only a handful caves in the karst belt of Puerto Rico, and we have a very incomplete understanding of cave use in this species. Though our analysis only included two cave and two surface populations and a limited number of nuclear markers, we have established a baseline from which to add additional markers and to collect genetic data from other cave populations as they are discovered. Importantly, we have provided evidence for a high level of genetic diversity in cave populations, and hence we urge that cave populations should be managed to ensure that the cave and the surrounding habitat remain intact. Furthermore, we suggest that movement of boas by people (an extremely common practice) is not likely to disrupt any existing mtDNA phylogeographic structure, though we would still discourage the practice. Boas are frequently removed from urban areas by wildlife officers and translocated to protected areas on other parts of the island (authors, pers. ob.). For example, between January and March 2012 at least nine boas were brought to a holding facility at Cambalache State Forest and then released in nearby protected areas. The origins of these boas are often unclear, because no data recording system is in place, but they could potentially be brought to Cambalache from anywhere on the island. Future work should explicitly evaluate whether this practice is harmful to local populations owing to the potential for outbreeding depression [39]. Though our results are preliminary, we have provided important information regarding the genetic diversity of behaviorally differentiated populations of this species; however, we strongly encourage the development of additional genetic markers for this species and the collection of genetic data from additional populations.
